# Pediatric Upper Extremity Firearm-related Injuries: A Level I Pediatric Trauma Center Experience

**DOI:** 10.5811/westjem.29333

**Published:** 2025-10-09

**Authors:** Ann Carol Braswell, Edgar Soto, Andrew D. Bloom, Eric Jorge, Erin F. Ransom, Rachel E. Aliotta

**Affiliations:** * University of Alabama at Birmingham and Children’s of Alabama Hospital, Department of Orthopedic Surgery, Birmingham, Alabama; † Johns Hopkins University, Department of Plastic Surgery, Baltimore, Maryland; ‡ University of Alabama at Birmingham, Department of Emergency Medicine, Birmingham, Alabama; § Children’s of Alabama Hospital, Department of Pediatric Emergency Medicine, Birmingham, Alabama

## Abstract

**Introduction:**

Firearm injuries have become increasingly more common in the pediatric population; however, there is a paucity of literature examining the management of these pediatric firearm-related injuries (FRI) specifically as they affect the upper extremity. This study identifies demographic and environmental risk factors in pediatric upper extremity FRIs and evaluates the severity of injury, concomitant injuries, and rates of surgical intervention in pediatric patients treated at a Level I pediatric trauma center over 20 years.

**Methods:**

We completed a retrospective analysis on 540 patients <18 years of age with FRIs at a single institution from 2001 – 2020. Of these, 72 (13%) had FRIs involving the upper extremity. The patients were stratified into groups based on whether they had received operative intervention or a bedside procedure for their injury and on their year of presentation between two decades (2001 – 2010 vs. 2011 – 2020). We obtained upper extremity injury-specific variables along with hospital demographics. The primary outcomes in this study included hospital length of stay, number of bullet wounds, motor and sensory deficits, and amputation.

**Results:**

In the last 10 years, the rate of upper extremity FRIs observed in the pediatric population has increased by 380% at our institution (15 vs. 57, *P* < .001). After 2010, cases were more likely to present with an increased number of gunshot wounds per patient (1.14 vs. 1.98, 95% confidence interval [CI] −0.94 – 0.24, *P* = .03) but were less likely to require admission to the intensive care unit (19% vs. 67%, *P* < .001). When stratifying by intervention, both the operative intervention and bedside procedure groups had a similar number of gunshot wounds (1.86 vs 1.76, 95% CI −0.52 – 0.43, *P* = .86). The operative intervention group was more likely to have had a soft tissue injury (68% vs. 35%, *P* = .005) and motor deficit at follow-up (45% vs.15%, *P* =.02). Patients in the operative intervention group had longer lengths of stay (9.66 vs. 2.25 days, 95% CI −1.16 – −0.21, *P* < .01) and more morbid injuries despite similar patient demographics.

**Conclusion:**

In the last decade, an increased frequency of pediatric upper extremity firearm-related injuries was noted despite a stagnant state population. Emphasis should continue to be placed on education and improving firearm safety in settings in which children are present.

## INTRODUCTION

Hand and upper extremity injuries are one of the leading causes of presentation to trauma and emergency departments in the pediatric population.^1^ Injury to the upper extremities can impair joints, bones, neurovascular structures, and the surrounding soft tissues leading to substantial morbidity and loss of function.^2^ Previously, firearm-related injuries (FRI) in children were reported rarely; however, in recent years pediatric firearm injuries have become increasingly more common.^3^,^4^ Firearm-related injuries in the pediatric population can cause disparate damage to both soft tissue and bone in children due to their small body habitus and lead to complex injury patterns and presentations.^4^

Using data from recent years, the Government Accountability Office estimates the initial hospital cost of FRI in the United States (in both pediatric and adult populations) to be over one billion dollars annually.^5^ Although the pediatric population accounts for only a small percentage of these injuries, further studies have shown that the economic burden of these injuries can be substantial.^6^–^8^ Pediatric FRIs have been associated with higher hospital costs and resource utilization than motor vehicle collisions, and a study done by Bongiorno et al found the average cost of FRIs among children in the US to be approximately 45,000 dollars per patient.^8^,^9^ Despite the increased incidence of FRIs in children, the literature describing the patterns or morbidity of FRIs to the hand and upper extremities in children is sparse and inconsistently presented.

Several studies have examined FRIs in the pediatric population at large. These studies often use national data repositories or samples, without granular or center-specific clinical data examining these firearm injuries as it relates to the hand or musculoskeletal system in children.^10^–^12^ This is the opposite of what we find reviewing the more extensive literature available regarding upper extremity firearm injuries in the adult population. In the context of adult firearm injuries, males are more affected by both fatal and nonfatal FRIs than females, with Black, young adult males being the most likely to sustain non-fatal wounds to the upper extremity.^13^,^14^ Firearm-related injuries to the hands also appear to be a common site of injury and much less likely to require hospitalization than a more proximal location.^14^ Therefore, a review and analysis of the pediatric population affected by FRIs to the hand and upper extremity remains necessary.

In this retrospective cohort study, we characterized upper extremity firearm injuries in children to describe the impact associated with management of these injuries. We sought to identify the differences in epidemiology and descriptive characteristics of pediatric firearm injuries over the last 20 years at our state’s only Level I pediatric trauma and tertiary care referral center. We hypothesized that there would be a substantial increase in the complexity and incidence of firearm-related injuries over the last 20 years in children.

## METHODS

### Study Design

**Figure 1:**
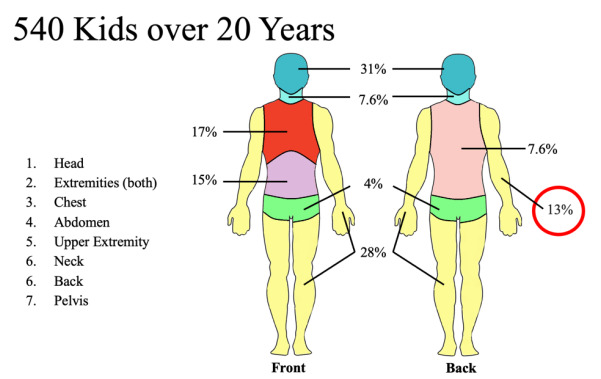
Firearm-related upper extremity injury vs injury to other parts of the body at a Level I pediatric trauma center.

We completed an institutional review board-approved, retrospective analysis on all patients <18 years of age with FRIs to the upper extremity treated at the only Level I pediatric hospital in the state of Alabama between January 1, 2001–December 31, 2020. We employed elements of optimal chart review discussed by Worster and Bledsoe.^15^ The abstractors were trained and used data abstraction forms to collect data. Inclusion/exclusion criteria as well as variables were defined prior to case selection. Inclusion criteria included <18 years of age and the mechanism of injury being firearm-related (ie, ballistic). We collected data via electronic health record by reviewing all subsequent inpatient and outpatient charts for each individual patient. From the 20-year database of over 540 firearm-injured pediatric patients (including children with all sustained FRIs) we identified 72 with upper extremity involvement (Figure 1). The 72 patients were then stratified based on whether they had received operative intervention (OR group) or bedside procedure (BP group) for their upper extremity injury, and by the year of presentation (January 1, 2001 – December 31, 2010 vs January 1, 2011 – December 31, 2020). The OR group was classified as the need for surgery at the time of initial presentation.

**Population Health Research Capsule**

What do we already know about this issue?

*Unintentional firearm related injury is the fourth leading cause of death among infants (age <1 yr) and is the number one cause of death among children and teens (aged 1–17yrs).* *24*

What was the research question?

*This study sought to identify whether there was a significant decade-interval increase in firearm related upper extremity injuries, with corresponding demographic and environmental risk factors.*

What was the major finding of the study?

*In the last 10 years, the rate of upper extremity firearm related injuries observed in the pediatric population has increased by 380% at our institution (15 vs. 57, P < .001).*

How does this improve population health?

*In the last decade, an increased frequency of pediatric upper extremity firearm-related injuries was noted despite a stagnant state population. Unintentional firearm injury deaths of children are preventable.*

### Primary Variables and Outcomes of Interest

Demographic variables obtained included age, sex, race/ethnicity, insurance status, and ZIP code/county. Race data were categorized as Black, White, Hispanic and other. Geographical data, based on patient residence, was broadly collected and then categorized as Jefferson County (the county where Children’s of Alabama is located), or other. Additional information included the following: when (season, month, year) and where (inside vs outside the home) the shooting occurred; intentionality of shooter; shooter identification (father, mother, self, etc); shooting classification (bystander, intruder, gun cleaning, etc); number of gunshot wounds; gun type; and storage reported (locked vs unlocked). We obtained medical data regarding the upper extremity injury (ie. structures involved, degree of injury) along with hospital demographics (ie, length of stay [LOS]/admission, procedures performed in the operating room vs bedside), number of follow-ups, and overall clinical outcomes, if available. The primary outcomes in this study included hospital LOS, number of bullet wounds, motor and sensory deficits, and amputation.

### Statistical/Analytical Approach

We reported descriptive statistics as means ± standard deviation for continuous variables and frequency with percentage for categorical variables. We assessed differences in outcomes between groups using two-sided independent *t*-tests for continuous variables and χ^2^ tests for categorical variables. All statistical significance was referenced to *P*-values of < .05. Because we attempted to evaluate as many cases as were available in the EHR, no power analysis was performed.

## RESULTS

**Figure 2:**
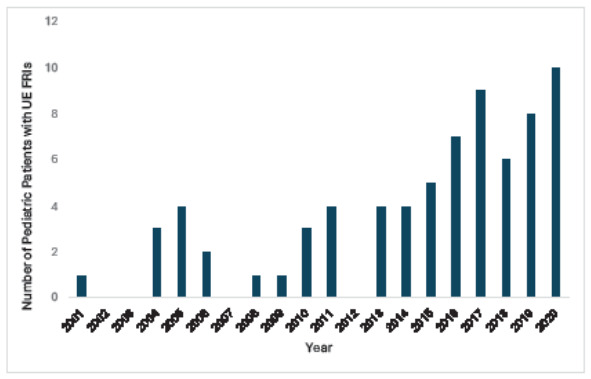
Pediatric upper extremity firearm-related injuries by year at our institution. *UE*, upper extremity; *FRI*, firearm-related injuries.

A total of 72 patients were included in this study: 15 patients from 2001–2010 and 57 patients from 2011 – 2020 (Table 1). The cohort had an average age of 11.7 years with most being male (69%), Black (83%), and insured by Medicaid (54%). In the 10-year period 2011 – 2020, the incidence of gunshot wounds to the upper extremity in the pediatric population increased by 380% at our institution (15 vs. 57, *P* < .001) (Figure 2). Despite this substantial increase in volume, the age (11.7 ± 5.0), race (predominantly Black), and insurance status did not change (*P* > .05). After 2010, cases were more likely to present with an increased number of gunshot wounds per patient (1.1 vs. 2, *P* = .03). A higher percentage of these patients were taken to the OR; however, that difference was not significant (47% vs. 54%, *P* = .59). Those patients presenting between 2011–2020 did experience a significantly shorter length of hospitalization (4.5 vs. 12.5 days *P* = .01) and were less likely to be admitted to the pediatric intensive care unit (19% vs. 66% P < .001) than the 2001 – 2010 group. When stratifying by operative intervention, the 38 children in the OR group had similar general demographics and a similar number of gunshot wounds when compared to the 34 in the bedside procedure group (1.9 vs 1.8; *P* = .86) (Table 2). The OR group was more likely to have an accompanying bony injury (85%), tendon injury (24%), soft tissue injury (68%), shoulder-joint FRI (21%), and motor deficit at follow-up (50%) than the BP group (*P* < .05). The OR groups also participated in significantly more occupational therapy (46% vs. 15% *P* = .02) than the BP group. The BP group was less likely to be admitted to the hospital for more than 24 hours and, when admitted, experienced significantly shorter hospital LOS (9.7 vs. 2.3 days *P* = .01). Significantly more patients in the BP group were shot inside their primary residence; however, there was no difference between groups in the known presence of firearms inside the home (*P* = .28) or in intention (*P* = .32).

When looking at the entire cohort, only two patients lost a limb/digit, 31% had motor deficits, and 16% had sensory deficits at follow-up. Black children were more likely to have non-upper extremity concomitant injuries (41.3% vs. 9.1% *P* = .04) and be a victim of intentional FRIs (incidence 6% vs. 9.1% in White children, *P* < .001). Blacks also presented with a significantly higher number of gunshots per patient (1.0 vs. 2.0 *P* = .003) Patients from Jefferson County (in which a large metropolitan population resides and where the pediatric trauma center itself is located) were more likely to have worse injuries involving the wrist, arterial injury, tendon injury, and retained bullets (*P* < .05).

## DISCUSSION

Firearm injury among children and teenagers in the US remains an area of significant public health concern with the rate of firearm deaths in this population steadily increasing.^16^ Children with non-fatal firearm injuries experience high operative and readmission rates and suffer long-term morbidity and mental health sequelae.^17^–^19^ Despite the magnitude of this public health and surgical crisis the morbidity of firearm injuries remains understudied. Here we identified a dramatic shift and increased frequency of FRIs to the upper extremity in pediatric trauma patients: of the 72 total pediatric cases identified with upper extremity injuries over the 20-year study period, 79% patients presented after 2010, and they also presented with an increase in the number of bullet wounds sustained. That said, following 2010, patents were less likely to get admitted to the PICU or to any floor for > 24 hours, potentially indicating an overall shift to outpatient-based follow-up for these injuries.

As the only pediatric Level I trauma center in Alabama, our hospital has a large number of injured children transferred to us for care. However, one critique of this review, in our experience, is that some minor gunshot wounds sustained in patients who live in remote or distant areas may have been treated locally and remain unaccounted for. Neither did this study include the Jefferson County coroner’s reports for those children who did not make it to the hospital ED during this 20-year period. Most patients in our study had Medicaid insurance, which supports recent evidence suggesting a higher frequency of pediatric firearm injuries in those with government-assisted payment, followed by private and uninsured patients. In this group, Black children and teenagers comprised 83% of the pediatric population affected by upper extremity firearm injuries. Considering that Blacks make up only 42% of the pediatric population in Jefferson County (and 44% in the state of Alabama), the large numbers of Black children and teenagers in our injured cohort represents a disparate level of exposure to gun violence among these youths.

While we were unable to characterize the societal cost of pediatric firearm injury in our study due to the retrospective nature of collection across two decades, several groups have estimated that the median cost of pediatric firearm-related hospitalizations is greater than $45,000 per patient event.^8^,^9^ While only two patients lost a digit in our cohort, over 31% had some form of documented motor deficit and 16% had sensory deficits. This long-term follow-up is severely limited due to loss of follow-up with time as patients often live far away and were flown in by air for their initial treatment. Naturally, it is difficult to quantify the long-term social and emotional impact on patients, families, and communities of this compromised daily function. This is concordant with accepted incidences in the literature, which show that while most victims of firearm-related violence survive their injuries, about half of children remain with a functional deficit.

Our findings of primarily male and non-White victims of pediatric firearm injuries mirror recent studies in other high-volume centers.^20^–^23^ These studies include those by Summers et al who looked at pediatric upper extremity injuries secondary to non-ballistic firearms; Nichols et al who analyzed demographics and mechanisms of injury for upper-extremity pediatric firearm injuries at one tertiary trauma center in Florida; Dabash et al who examined characteristics and outcomes of 10 pediatric upper-extremity FRIs cases at a trauma center on the US-Mexican border; and Tarkunde et al who looked at epidemiological factors and management of FRIs to the wrist and hand in 29 pediatric patients (and 220 adult patients) at a Level I trauma center. Dabash found males to be more affected than females and most injuries due to either violence (60%) or accidental discharge (40%). Of note, none of the patients analyzed by Dabash et al had lasting physical deficits as a result of their injury.^22^ On the other hand, Nichols et al found the hand to be the most frequent location of Andinjury, with factors including male sex, White-not Hispanic or Latino race/ethnicity, and adolescent age contributing to an increased risk for an upper extremity injury.^21^

## LIMITATIONS

This study has several limitations that warrant consideration. This was a single-center study, thus limiting its external validity. The single hospital location, with exclusion of those patients treated at outside facilities, may have selectively biased the data to more severe injuries that required treatment at a Level I trauma center and limit the generalizability of this data to other communities. Similarly, the true frequency of pediatric upper-extremity firearm injuries is likely still under-reported. In cases of isolated hand or minor injury, many patients may have been treated in a local ED or transported across state lines to an adjacent pediatric hospital if it was closer. That said, because our center is a high-volume pediatric and academic-trauma center, we believe that these injuries are more often identified here than in other centers. However, based on our significant findings, prospective database-reporting studies would be informative for guiding additional recommendations for diagnosis and management of pediatric upper-extremity firearm injuries.

Despite its limitations, this study sheds light on the adverse outcomes sustained by children as a result of upper extremity injuries from firearms. The size of our patient population is significantly larger than that of previous studies examining firearm injuries in children. Our study’s specific focus on firearm-related musculoskeletal injuries over a 20-year span provides a long timeframe for evaluation, and we were able to present data about adverse outcomes from these injuries with at least a minmum three-year follow-up from the last patient collected at the time of our study. Our finding of increased risk of adverse outcomes in Black males and Medicaid/uninsured presents a focused population for gun safety outreach and future community health interventions. Continued re-evaluation of firearm injury prevention strategies and targeted efforts at reducing gun violence in our communities may be beneficial in ensuring continued decline in firearm-related injuries among America’s youth.

## CONCLUSION

In the decade 2011–2020, pediatric patients suffered increased morbidity from their sustained firearms-related injuries, representing a significantly increased frequency of firearm-related injuries to the upper extremity. Given the importance of initial patient management and long-term care, clinicians are encouraged to assess for, document, and manage upper-extremity firearms injuries in pediatric trauma patients and consult orthopedic surgeons and plastic surgeons when appropriate. Future prospective studies are necessary to better characterize upper extremity patient-injury patterns and outcomes to generate practice-guiding recommendations for this patient population.

## Figures and Tables

**Table 1:** Demographics of pediatric upper extremity firearm related injuries stratified by decade of presentation

	2001–2010 (n=15)	2011–2020 (n=57)	Total (N=72)	P-value
Male sex	8 (53%)	42 (74%)	50 (69%)	.23
Race/ethnicity				.68
Black	13 (87%)	46 (81%)	59 (82%)	
White	2 (13%)	10 (18%)	12 (17%)	
Age at injury	11.45 (5.07)	11.72 (5.05)	11.67 (5.02)	.85
Insurance				.78
Medicaid	8 (53%)	31 (54%)	39 (54%)	
Private	4 (27%)	11 (19%)	15 (21%)	
None	3 (20%)	15 (26%)	18 (25%)	
Number of gunshot wounds	1.14 (0.36)	1.98 (2.65)	1.81 (2.39)	.03
Required operative intervention	7 (47%)	31 (54%)	38 (53%)	.59
Non-upper extremity concomitant injury	9 (60%)	13 (23%)	22 (31%)	.02
Injury location				
Neck/plexus	4 (27%)	2 (4%)	6 (8%)	< .01
Scapula/clavicle	2 (13%)	1 (2%)	3 (4%)	.05
Shoulder	3 (20%)	6 (11%)	9 (13%)	.32
Upper arm	5 (33%)	23 (40%)	28 (39%)	.62
Elbow	1 (7%)	8 (14%)	9 (13%)	.44
Lower arm	1 (7%)	10 (18%)	11 (15%)	.30
Wrist	1 (7%)	5 (9%)	6 (8%)	.79
Hand/digits	3 (20%)	15 (26%)	18 (25%)	.62
Nerve injury	5 (33%)	8 (14%)	13 (18%)	.08
Artery injury	4 (21%)	3 (5%)	6 (8%)	.01
Bone injury	6 (40%)	32 (56%)	38 (53%)	.27
Tendon injury	3 (20%)	7 (12%)	10 (14%)	.44
Soft tissue injury	8 (53%)	30 (53%)	38 (53%)	.96
No bullet retained (In/out)	3 (20%)	17 (30%)	20 (8%)	.45
Hand injury specific clinic f/u	8 (53%)	32 (56%)	40 (56%)	.17
Motor deficit	4 (27%)	18 (32%)	22 (31%)	.92
Sensory deficit	2 (13%)	9 (16%)	11 (16%)	.97
Loss of limb/digit	0 (0%)	2 (4%)	2 (3%)	.75
Shot inside primary residence	8 (53%)	27 (47%)	35 (49%)	.87
Shooter Identification				.35
Unknown	6 (40%)	32 (56%)	38 (53%)	
Friend	3 (20%)	5 (9%)	8 (11%)	
Father	1 (7%)	4 (7%)	5 (7%)	
Mother	0 (0%)	0 (0%)	0 (0%)	
Sibling	2 (13%)	4 (7%)	6 (8%)	
Self	2 (13%)	8 (14%)	10 (14%)	
Other	1 (7%)	4 (7%)	5 (7%)	
Gun type				.14
Shotgun	1 (7%)	0 (0%)	1 (1%)	
Handgun	5 (33%)	33 (58%)	38 (53%)	
Long gun	3 (20%)	7 (12%)	10 (14%)	
Assault-style	0 (0%)	2 (4%)	2 (3%)	
Unknown	6 (40%)	15 (26%)	21 (29%)	
Intentional	8 (53%)	27 (47%)	35 (49%)	.87
Weapon unlocked at time of injury	3 (21%)	20 (35%)	23 (32%)	.58
Classification of injury encounter				.36
Bystander	1 (7%)	12 (21%)	13 (18%)	
Intruder	1 (7%)	0 (0%)	1 (1%)	
Cleaning gun	0 (0%)	1 (2%)	1 (1%)	
Drive-by	2 (13%)	8 (14%)	10 (14%)	
Homicide	4 (27%)	12 (21%)	16 (22%)	
Police involvement	1 (7%)	0 (0%)	1 (1%)	
Play	4 (27%)	13 (23%)	17 (24%)	
Suicide	0 (0%)	1 (2%)	1 (1%)	
Unknown	2 (13%)	10 (18%)	6 (8%)	
Discharged from ED	0 (0%)	19 (33%)	19 (26%)	.01
Observe < 24 hrs	2 (13%)	10 (18%)	12 (17%)	.70
Admit (> 24 hrs)	13 (87%)	27 (47%)	40 (56%)	.01
Admit PICU	10 (67%)	11 (19%)	21 (29%)	<.001
# Hospital days	12.53 (10.52)	4.48 (11.02)	6.16 (11.34)	0.01

*ED*, emergency department; *f/u*, follow-up; *PICU*, pediatric intensive care unit.

**Table 2:** Patient characteristics and outcomes by the operative-intervention group vs bedside-procedure group.

	OR group (n=38)	BP group (n=34)	Total (N=72)	P-value
Male sex	22 (58%)	28 (82%)	50 (69%)	.07
Race/ethnicity				.71
Black	31 (82%)	28 (85%)	59 (83%)	
White	7 (18%)	5 (15%)	12 (17%)	
Age at injury	11.17 (5.67)	12.22 (4.19)	11.67 (5.02)	.38
Insurance				.54
Medicaid	22 (58%)	17 (50%)	39 (54%)	
Private	6 (16%)	9 (27%)	15 (21%)	
None	10 (26%)	8 (24%)	18 (25%)	
Number of gunshot wounds	1.86 (2.36)	1.76 (2.46)	1.81 (2.39)	.86
Non-upper extremity concomitant Injury	14 (37%)	8 (24%)	22 (31%)	.27
Injury location				
Neck/plexus	2 (5%)	4 (12%)	6 (8%)	.32
Scapula/clavicle	2 (5%)	1 (3%)	3 (4%)	.62
Shoulder	2 (5%)	7 (21%)	9 (13%)	.05
Upper arm	14 (37%)	14 (41%)	28 (39%)	.71
Elbow	7 (18%)	2 (6%)	9 (13%)	.11
Lower arm	8 (21%)	3 (9%)	11 (15%)	.15
Wrist	5 (13%)	1 (3%)	6 (8%)	.12
Hand/digits	11 (29%)	7 (21%)	18 (25%)	.41
Nerve injury	6 (16%)	7 (21%)	13 (18%)	.60
Artery injury	5 (13%)	1 (3%)	6 (8%)	.18
Bone injury	28 (74%)	10 (26%)	38 (53%)	<.001
Tendon injury	9 (24%)	1 (3%)	10 (14%)	.01
Soft tissue injury	26 (68%)	12 (35%)	38 (53%)	.01
No bullet retained (In/out)	5 (13%)	15 (44%)	20 (28%)	<.001
Hand injury specific clinic f/u	26 (68%)	14 (41%)	40 (56%)	.07
Motor deficit	17 (45%)	5 (15%)	22 (31%)	.02
Sensory deficit	5 (15%)	6 (18%)	11 (16%)	.86
Loss of limb/digit	2 (5%)	0 (0%)	2 (3%)	.40
OT documented	16 (42%)	5 (15%)	21 (30%)	.02
Season shooting occurred				.31
Fall	7 (18%)	8 (24%)	15 (21%)	
Spring	6 (16%)	5 (15%)	11 (15%)	
Summer	13 (34%)	17 (50%)	30 (42%)	
Winter	12 (32%)	4 (12%)	16 (22%)	
Shot inside primary residence	11 (29%)	19 (56%)	30 (42%)	.01
Gun-owning home	18 (47%)	9 (27%)	27 (38%)	.28
Shooter Identification				.67
Unknown	17 (45%)	21 (62%)	38 (53%)	
Friend	4 (11%)	4 (12%)	8 (11%)	
Father	4 (11%)	1 (3%)	5 (7%)	
Mother	0 (0%)	0 (0%)	0 (0%)	
Sibling	4 (11%)	2 (6%)	6 (8%)	
Self	6 (16%)	4 (12%)	10 (14%)	
Other	3 (8%)	2 (6%)	5 (7%)	
Gun type				.08
Handgun	18 (47%)	20 (59%)	38 (53%)	
Long gun	7 (18%)	3 (9%)	10 (14%)	
Assault-style	2 (5%)	0 (0%)	2 (3%)	
Unknown	10 (26%)	11 (32%)	21 (29%)	
Intentional	17 (45%)	18 (53%)	35 (49%)	.32
Weapon unlocked at time of injury	14 (37%)	9 (27%)	23 (32%)	.03
Shooting classification				.15
Bystander	4 (11%)	9 (27%)	13 (18%)	
Intruder	1 (3%)	0 (0%)	1 (1%)	
Cleaning gun	0 (0%)	1 (3%)	1 (1%)	
Drive-by	5 (13%)	5 (15%)	10 (14%)	
Homicide	9 (24%)	7 (21%)	16 (22%)	
Police involved	0 (0%)	1 (3%)	1 (1%)	
Play	10 (26%)	7 (21%)	17 (24%)	
Suicide	1 (3%)	0 (0%)	1 (1%)	
Unknown	8 (21%)	4 (12%)	12 (17%)	
Discharged from ED (Observed < 23 hrs.)	3 (8%)	16 (47%)	19 (26%)	<.001
Admit (> 24 hrs.)	32 (84%)	8 (24%)	40 (56%)	<.001
Admit PICU	16 (42%)	5 (15%)	21 (29%)	0.01
# Hospital days	9.66 (13.79)	2.25 (5.77)	6.16 (11.34)	0.01

*OR*, operative procedure; *BP*, bedside procedure; *OT*, occupational therapy; *ED*, emergency department; *f/u*, follow-up; *PICU*, pediatric intensive care unit.

## References

[1] Lee A, Colen DL, Fox JP (2021). Pediatric hand and upper extremity injuries presenting to emergency departments in the United States: epidemiology and health care-associated osts. Hand (N Y).

[2] Ng ZY, Askari M, Chim H (2015). Approach to complex upper extremity injury: an algorithm. Semin Plast Surg.

[3] Veenstra M, Patel V, Donoghue L (2015). Trends in pediatric firearm-related injuries over the past 10 years at an urban pediatric hospital. J Pediatr Surg.

[4] Carter CW, Sharkey MS, Fishman F (2017). Firearm-related musculoskeletal injuries in children and adolescents. J Am Acad Orthop Surg.

[5] United Sates Government Accountability Office (2021). FIREARM INJURIES Health Care Service Needs and Costs.

[6] Taylor JS, Madhavan S, Han RW (2021). Financial burden of pediatric firearm-related injury admissions in the United States. PLoS One.

[7] Sidhu S, Mandelbaum A, Dobaria V (2021). National trends in the cost burden of pediatric gunshot wounds across the United States. J Pediatr.

[8] Fraser Doh K, Sheline E, Wetzel M (2021). Comparison of cost and resource utilization between firearm injuries and motor vehicle collisions at pediatric hospitals. Acad Emerg Med.

[9] Bongiorno DM, Badolato GM, Boyle M (2021). United States trends in healthcare charges for pediatric firearm injuries. Am J Emerg Med.

[10] Fowler KA, Dahlberg LL, Haileyesus T (2017). Childhood firearm injuries in the United States [published correction appears in. Pediatrics.

[11] Allareddy V, Nalliah RP, Rampa S (2012). Firearm related injuries amongst children: estimates from the nationwide emergency department sample. Injury.

[12] Lee J, Moriarty KP, Tashjian DB (2013). Guns and states: pediatric firearm injury. J Trauma Acute Care Surg.

[13] Hooper RC, Shauver MJ, Chou CH (2021). Epidemiology of upper extremity firearm injuries among major trauma hospitals in the United States. Plast Reconstr Surg.

[14] Toston RJ, Graf AR, Dawes AM (2023). Upper extremity firearm injuries: epidemiology and factors predicting hospital admission. Eur J Orthop Surg Traumatol.

[15] Worster A, Bledsoe RD, Cleve P (2005). Reassessing the methods of medical record review studies in emergency medicine research. Ann Emerg Med.

[16] Roberts BK, Nofi CP, Cornell E (2023). Trends and disparities in firearm deaths among children. Pediatrics.

[17] Evans PT, Pennings JS, Samade R (2020). The financial burden of musculoskeletal firearm injuries in children with and without concomitant intra-cavitary injuries. J Pediatr Surg.

[18] Phillips R, Shahi N, Bensard D (2020). Guns, scalpels, and sutures: the cost of gunshot wounds in children and adolescents. J Trauma Acute Care Surg.

[19] Koenig SM, Russell RT (2023). Pediatric firearm injury: defining the full spectrum. Ann Surg.

[20] Summers AR, Cheema AN, Pirruccio K (2022). Epidemiology of pediatric nonballistic firearm injuries to the upper extremity in the United States from 2000 to 2017. Hand (N Y).

[21] Nichols DS, Audate M, King C (2021). Pediatric upper extremity firearm injuries: an analysis of demographic factors and recurring mechanisms of injury. World J Pediatr.

[22] Dabash S, Gerzina C, Simson JE (2018). Pediatric gunshot wounds of the upper extremity. Int J Orthop.

[23] Tarkunde YR, Clohisy CJ, Calfee RP (2023). Firearm injuries to the wrist and hand in children and adults: an epidemiologic study. Hand (N Y).

[24] Wilson RF, Mintz S, Blair JM, US Department of Health and Human Services: Centers for Disease Control and Prevention (2023). Unintentional Firearm Injury Deaths among children and adolescents aged 0-17—national violent death reporting system, US, 2003–2021. MMWR.

